# Un kyste hydatique osseux: une localisation rare au niveau de l’os iliaque

**DOI:** 10.11604/pamj.2016.24.226.6322

**Published:** 2016-07-13

**Authors:** Yassine Nhamoucha, Othmane Alaoui, Aliou Doumbia, Mohammed Oukhoya, Hicham Abdellaoui, Mohammed Tazi, Lamyae Chater, Karima Atarraf, Mounir Arroud, Abderahman Afifi

**Affiliations:** 1 Service de Traumato-Orthopédie Pédiatrique, Hôpital Mère et Enfant, CHU de Fès, Maroc; 2 Service de Chirurgie Pédiatrique, CHU Hassan II, Fès, Maroc

**Keywords:** Hydatidose, chirurgie, os iliaque, Hydatid disease, surgery, hip bone

## Abstract

L'hydatidose est une affection parasitaire liée au développement chez l'homme de la forme larvaire d'un cestode, à savoir un tænia de très petite taille dénommé Echinococcus Granulosus. Cette anthropozoonose présente une diversité de formes anatomoradiologiques lié aux nombreux aspects topographiques et évolutifs des kystes. L'hydatidose osseuse est rare, elle ne représente que 0,9 à 2,5% de l'ensemble des localisations. Nous rapportons l'observation d'un enfant de 9 ans, qui a été admis chez nous pour une boiterie fébrile avec une masse au niveau de la fosse iliaque droite, révélant un kyste hydatique au dépend de l'os iliaque. Le bilan lésionnel avait objectivé un kyste hydatique de l'os iliaque avec extension aux parties molles adjacentes. Le traitement chirurgical conclu a un kyste surinfecté d'ou la réalisation d'une exérèse chirurgicale du kyste avec drainage. L'ostéopathie hydatique est infiltrante, diffuse, lente et progressive, ce qui rend le diagnostic tardif et qui compromet la qualité du traitement.

## Abstract (en)

Hydatid disease is a parasitic disease caused by the development in humans of the larval form of a tapeworm, namely a very small tænia called Echinococcus Granulosus. This anthropozoonosis is characterized by the presence of different types of anatomo-radiologic variants associated with various topographic and evolutionary aspects of the cysts. Bone hydatid disease is a rare condition, it accounts for only 0.9-2.5% of all locations. We report the case of a 9 year old child, who was admitted with febrile lameness and with a mass in the right iliac fossa, revealing a hydatid cyst at the level of the hip bone. Lesion assessment objectified a hydatid cyst of the hip bone with extension into adjacent soft tissues. An infected cyst was detected during surgery, hence the performance of a surgical excision of the cyst with drainage. Hydatic osteopathy is infiltrating, diffuse, slow and gradual, causing delays in diagnosis and compromising the quality of care.

## Introduction

Le kyste hydatique est une affection parasitaire assez fréquente dans les pays d'endémie. Il siège fréquemment au niveau hépatique et pulmonaire. L'échinococcose osseuse est rare: sa fréquence varie de 1 à 2%. Elle est caractérisée par une latence clinique longue: l'infestation peut se faire dans l'enfance et c'est à l'âge adulte que l'affection est découverte [1, 2]. Nous rapportons un cas exceptionnel de kyste hydatique isolé de l'os iliaque en illustrant l'apport de l'imagerie dans le diagnostic et la difficulté thérapeutique de cette affection.

## Patient et observation

**Figure 1:**
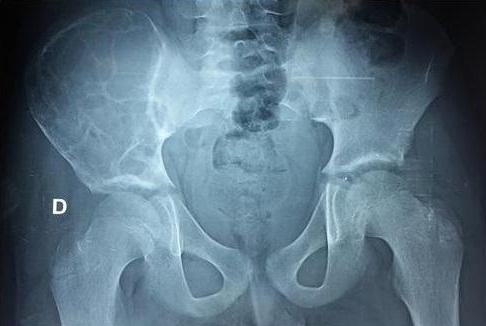
Radiographie standard du bassin de face montrant le plus souvent des images lytiques aréolaires mal limitées, réalisant l’aspect classique en « nid d’abeille ». Il n’existe ni réaction périostée ni décalcification régionale

**Figure 2:**
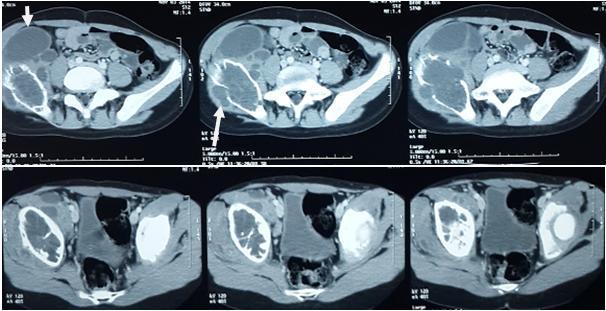
Coupe scannographique objectivant l’atteinte osseuse au niveau iliaque avec présence de formation kystique intrapelvienne (flèches)

**Figure 3:**
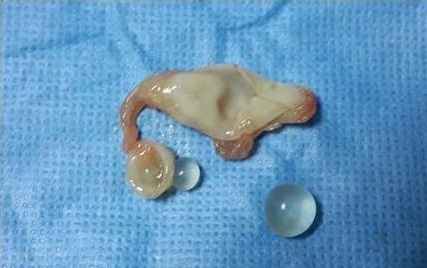
Image postopératoire montrant une membrane proligère du kyste hydatique avec des vésicules filles

**Figure 4:**
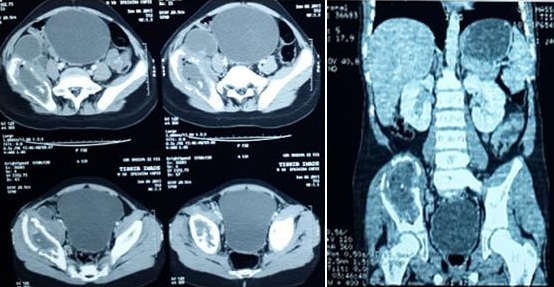
Coupe scannographie objectivant le même aspect préopératoire avec une légère régression de la taille des collections

Il s'agit de l'enfant IMAD, âgé de 11 ans, 2^ème^ d'une fratrie de 3, sans antécédents pathologiques notables, qui a consulté aux urgences pédiatriques pour une boiterie non fébrile avec une tuméfaction douloureuse au niveau de la fosse iliaque droite, ayant progressivement augmenté de volume depuis un mois. L'examen clinique à l'admission trouvait un enfant stable sur le plan hémodynamique et respiratoire avec une masse au niveau de la fosse iliaque droite douloureuse, faisant environ 6 centimètres de grand axe, fixe par rapport au deux plans. L'examen de la hanche droite était sans particularité. Le bilan infectieux a été positive (CRP à 192 mg/l, globules blancs à 22 000 éléments/m^3^). L'enfant a bénéficié d'une radiographie standard du bassin de face qui a montré des images lacunaires de taille variable, mal délimitées, sans réaction périostée (Figure 1). On a compléter le bilan par une sérologie hydatique qui était positive et une tomodensitométrie abdominopelvienne qui a objectivée un os iliaque essoufflé avec une rupture de sa corticale antérieure associé à plusieurs collections au niveau du muscles psoas arrivant jusqu'au niveau du muscle vaste médiale (Figure 2). La décision thérapeutique était d'aller évacuer ces collections avec un lavage abondant au sérum salé hypertonique (Figure 3). L'évolution a été marquée par la normalisation du bilan infectieux et l'assèchement des lames de delbet, d'où la réalisation d'un scanner de contrôle à j 10 qui a montrer presque le même aspect avec une légère régression de la taille des collections (Figure 4). L'enfant a été repris avec un abord chirurgical large sur l'aile iliaque, on a procéder à un curetage aspiration, évacuation des collections et nettoyage abondant au sérum hypertonique. Le patient est toujours hospitalisé au service sous antibiothérapie associée à un traitement antihelminthique avec éventuel scanner de contrôle dans 10 jours.

## Discussion

Le kyste hydatique est dû à *Echinococcus granulosus* ou ténia échinococcique qui vit à l´état adulte dans l´intestin du chien, se localise préférentiellement au niveau pulmonaire (20 à 30%), et hépatique (60 à 70%) [3–5]. Elle sévit à l'état endémique particulièrement en Amérique du Sud, en Australie mais également dans le pourtour du bassin méditerranéen et en Europe Centrale [6]. La contamination osseuse se fait essentiellement par voie hématogène [7], mais une invasion osseuse secondaire à partir d'une atteinte primitive des parties molles est possible, l'ostéopathie hydatique est infiltrante, diffuse, lente et progressive avec de nombreuses microvésicules sans enkystement du parasite [2, 8]. Ce cas de localisation iliaque que nous rapportons est certainement primitif. Les signes cliniques révélateurs de l'affection ne sont pas spécifiques et dépendent de la localisation. Ils sont dominés par la douleur et la tuméfaction, comme chez notre patient. L'atteinte pelvienne concerne l'os iliaque dans 16,4% (16% pour Froment, 14,4% pour Devé). Elle est de mauvais pronostic fonctionnel par l'extension à l'articulation coxo-fémorale et plus rarement au sacrum [9, 10]. L'examen clinique est pauvre, il est marqué par la conservation de l'état général et l'apyrexie du patient. Une discrète boiterie à la marche est observée lorsque la parasitose siège aux membres inférieurs ou au bassin. La pression de la région concernée peut déclencher une douleur. L'examen neurologique peut mettre en évidence des signes d'irritation radiculaire ou pyramidale [1].

Sur le plan paraclinique, la radiographie standard reste l'examen de référence pour le diagnostic. Elle montre le plus souvent des images lytiques aréolaires mal limitées, réalisant l'aspect classique en « nid d'abeille » sans réaction périostée ni décalcification régionale [6]. L'intérêt de l'échographie est essentiellement pour explorer les parties molles à la recherche de l'abcès ossifluent. Elle contribue, de même que la radiographie du thorax, au bilan de la maladie hydatique, à la recherche de localisations viscérales associées pouvant orienter le diagnostic [11]. La TDM et l'IRM précisent l'atteinte osseuse, apprécient l'étendue locorégionale et constituent un excellent moyen de surveillance de l'évolution de la maladie [12].

Le traitement actuel de l'échinococcose osseuse est médicochirurgical [2]. Les buts du traitement médical sont la réduction de la taille des kystes, la stérilisation de leur contenu en préopératoire et en postopératoire pour traiter les petits kystes passes inaperçus [13]. Le traitement chirurgical consiste en une exérèse « carcinologique » des lésions qu'on assimile à une véritable tumeur maligne avec ablation complète des lésions hydatiques, mais malgré les différentes méthodes thérapeutiques les taux de rechutes après exérèse partielle sont très importants [3].

## Conclusion

L'hydatidose osseuse, reste une localisation rare, même en zone endémique comme le Maroc. Son tableau clinique pauvre et son évolution insidieuse sont responsable d'un retard diagnostic. L'imagerie médicale permet d'établir un bilan lésionnel précis pour planifier une large résection chirurgicale. Mais malgré tout les méthodes thérapeutiques l'éducation sanitaire dans les pays d'endémie restent les meilleures mesures permettant de limiter les dégâts considérables engendres par cette parasitose.
